# Risk Prediction Model for Chronic Kidney Disease in Thailand Using Artificial Intelligence and SHAP

**DOI:** 10.3390/diagnostics13233548

**Published:** 2023-11-28

**Authors:** Ming-Che Tsai, Bannakij Lojanapiwat, Chi-Chang Chang, Kajohnsak Noppakun, Piyapong Khumrin, Ssu-Hui Li, Chih-Ying Lee, Hsi-Chieh Lee, Krit Khwanngern

**Affiliations:** 1 Department of Emergency Medicine, School of Medicine, Chung Shan Medical University, Taichung 40201, Taiwan; terence39@yahoo.com; 2 Department of Emergency Medicine, Chung Shan Medical University Hospital, Taichung 40201, Taiwan; 3 Faculty of Medicine, Chiang Mai University, Chiang Mai 50200, Thailand; bannakij.lojana@cmu.ac.th; 4 School of Medical Informatics & IT Office, Chung Shan Medical University Hospital, Taichung 40201, Taiwan; changintw@gmail.com; 5 Department of Information Management, Ming Chuan University, Taoyuan 33348, Taiwan; 6 Division of Nephrology, Department of Internal Medicine, Faculty of Medicine, Chiang Mai University, Chiang Mai 50200, Thailand; kajohnsak.noppakun@cmu.ac.th; 7 Pharmacoepidemiology and Statistics Research Center (PESRC), Faculty of Pharmacy, Chiang Mai University, Chiang Mai 50200, Thailand; 8 Department of Family Medicine, Faculty of Medicine, Chiang Mai University, Chiang Mai 50200, Thailand; u4507075@hotmail.com; 9 Department of Computer Science and Information Engineering, National Quemoy University, Kinmen 89250, Taiwan; 10 College of Bioresources and Agriculture, National Taiwan University, Taipei 10663, Taiwan

**Keywords:** chronic kidney disease, random forest, SHAP, Thailand, artificial intelligence

## Abstract

Chronic kidney disease (CKD) is a multifactorial, complex condition that requires proper management to slow its progression. In Thailand, 11.6 million people (17.5%) have CKD, with 5.7 million (8.6%) in the advanced stages and >100,000 requiring hemodialysis (2020 report). This study aimed to develop a risk prediction model for CKD in Thailand. Data from 17,100 patients were collected to screen for 14 independent variables selected as risk factors, using the IBK, Random Tree, Decision Table, J48, and Random Forest models to train the predictive models. In addition, we address the unbalanced category issue using the synthetic minority oversampling technique (SMOTE). The indicators of performance include classification accuracy, sensitivity, specificity, and precision. This study achieved an accuracy rate of 92.1% with the top-performing Random Forest model. Moreover, our empirical findings substantiate previous research through highlighting the significance of serum albumin, blood urea nitrogen, age, direct bilirubin, and glucose. Furthermore, this study used the SHapley Additive exPlanations approach to analyze the attributes of the top six critical factors and then extended the comparison to include dual-attribute factors. Finally, our proposed machine learning technique can be used to evaluate the effectiveness of these risk factors and assist in the development of future personalized treatment.

## 1. Introduction

Chronic kidney disease (CKD) is an increasingly severe condition in today’s aging society [1,2,3]. Population aging and the associated higher hypertension increase the prevalence of hyperlipidemia and hyperglycemia, thus increasing the incidence of CKD. Snively, C. S. and Gutierrez, C. [4] discussed the management of the common complications of kidney disease, which, in the early stages, show few symptoms and are usually detected by accidental diagnosis during blood or urine tests. In the middle and late stages, symptoms such as fatigue, shortness of breath, nausea, lower extremity edema, hematuria, etc. may occur, and, as the illness increases, the kidneys may eventually lose their function completely [5,6,7]. Although the leading causes of CKD are mostly related to diabetes and hypertension, there are other common causes, such as glomerulonephritis, polycystic kidney disease, prenatal kidney and urinary tract abnormalities, or autoimmune diseases.

Nowadays, the number of people suffering from CKD in various countries is increasing year by year, and the problems faced by developing countries are not only the soaring number of CKD patients but also the hidden social external costs and public health issues. In 2020 in Thailand, 11.6 million people (17.5%) were reported to have CKD, of which about 5.7 million (8.6%) are already in the advanced stages, and >100,000 people require hemodialysis [8]. In today’s global village, once the global economy is launched, there can be mutual influence among different issues [9]. In the context of the medical domain, it is imperative to transcend regional boundaries and integrate technological analyses to foster human well-being. The objective of this study is to develop a risk prediction model for CKD in Thailand. Artificial intelligence, with various famous machine learning algorithms, namely IBK, Random tree, Decision Table, J48, and Random Forest, were utilized in the WEKA environment in this study. Our experimental results show that the proposed machine learning algorithms and SHAP technique can be used to evaluate the effectiveness of these risk factors and have the potential to develop personalized interventions and treatments in the future.

WEKA 3.8.6(Waikato Environment for Knowledge Analysis) is a popular suite of machine learning software platforms for data mining that provides a graphical user interface for data preprocessing, clustering, classification, regression, visualization, and association rule mining, as well as unsupervised and supervised learning with numerous applications in many fields including medical informatics. Kumar and Khatri [10] compared various accuracy metrics of WEKA classifiers, such as the true positive rate, false positive rate, precision, recall, and f-measure, to create an early disease classification based on medical data. Similarly, Kodati et al. [11] compared clustering algorithms in the WEKA tool to identify the best suited for a heart disease dataset. In addition, artificial intelligence has been applied to the interpretation of numerical and text-based clinical data [12,13] as well as the diagnosis of medical images [14,15,16].

Unbalanced datasets, such as those including medical data, create multiple challenges. To solve data imbalances, the synthetic minority oversampling technology (SMOTE) can be used. SMOTE is an oversampling algorithm the primary purpose of which is to amplify new minority samples across a sample in which research can create more simulations to reach a balance among the different types of specimens [17,18].

The Shapley additive explanations (SHAP) analysis derives from game theory. Different attributes and factors are compared to the players participating in the game. The prediction of the model analyzes the contribution of each player in the game and calculates the player factors’ Shapley value [19]. The Shapley value is an ideal set of attributes used to measure the attribute’s contribution to the prediction fairly and provide a common nonlinear model prediction method. In machine learning, training a model on a set of features and attributes interpreted as a player’s value function, the Shapley value also provides a natural way to calculate which features contribute to predictions to visualize the results using SHAP analysis [20].

The subsequent sections include the “Materials and Methods,” in which the methodologies employed in the study are elaborated, offering a transparent framework for the experimental approach. Following this, the “Results” section presents the findings and outcomes derived from the experiments conducted in this study. Subsequently, the “Discussion” section engages in an in-depth analysis and interpretation of the experimental results, delving into the implications and broader context of the study. Finally, the “Conclusion” section summarizes the significance of the research and suggests possible avenues for future exploration.

## 2. Materials and Methods

The dataset of this study included 17,100 patients with their associated disease factors from a Thai hospital-based CKD registry database. In this database, 14 attributes were recorded as clinical latent factors: sex, age, serum albumin, blood urea nitrogen (BUN), cholesterol, direct bilirubin, globulin, glucose, high-density lipoprotein cholesterol, low-density lipoprotein cholesterol (LDL), total bilirubin, total protein, triglyceride, and white blood cell count. Each of the instances is represented using a feature vector like {2, 63, 1, 2, 2, 1, 1, 1, 1, 2, 1, 1, 1, 1}, which indicates a patient with the corresponding attributes {sex:female, age:63, albumin:normal, BUN:abnormal, cholesterol:abnormal, direct bilirubin:normal, blobulin:normal, blucose:normal, HDL:normal, LDL:abnormal, total bilirubin:normal, totol protein:normal, triglyceride:normal, white blood cell count:normal}. In summary, the first element of the feature vector is 1 for males and 2 for females; the second element of the feature vector is the age; and the rest of the element is 1 if the corresponding value is normal and 2 if the corresponding value is abnormal.

**Figure 1:**
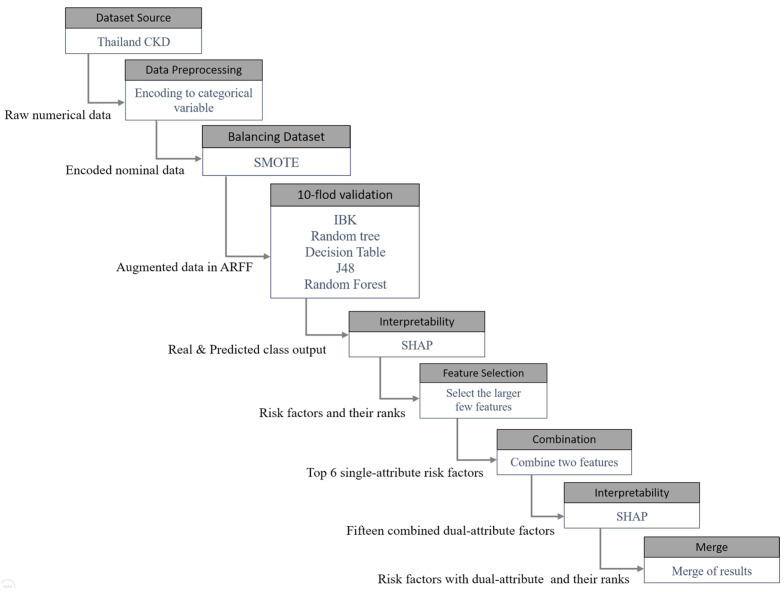
Experimental flow chart 2.1 data preprocessing.

The experimental procedure is presented in Figure 1. First, we included patients with a glomerular filtration rate (GFR) < 60 mL/min/1.73 m^2^ for over three months. Next, to improve the fit of the dataset with our machine learning algorithm and SHAP analysis, all categorical data were converted to numerical data. Then, due to the severe data imbalance between positive and negative cases, the SMOTE technique was applied to balance the dataset. To train the model’s predictions, 10-fold cross-validation was used. IBK, Random Tree, Decision Table, J48, and Random Forest were the algorithms to be compared in this study. Thereafter, SHAP analysis was used to interpret and analyze the importance and interaction of the features. The top six most significant terms were combined pairwise, and the same SHAP analysis results from the first stage were used. Finally, the results of both phases were combined.

### 2.1. Data Preprocessing

Data preprocessing plays a pivotal role in the realm of AI applications, acting as the essential foundation upon which accurate and efficient models are built. This crucial step involves cleaning, transforming, and organizing raw data to ensure its quality and relevance, as illustrated in [21,22]. By identifying and rectifying errors, handling missing values, and standardizing formats, data preprocessing enhances the overall integrity of the dataset. Moreover, data preprocessing aids in addressing issues like data imbalance and outliers, promoting the creation of robust and reliable AI models capable of handling diverse and real-world scenarios. In essence, the meticulous preparation of data through preprocessing lays the groundwork for the success of AI applications by fostering model accuracy, generalization, and overall performance.

#### 2.1.1. Data Conversion

To increase data suitability for machine learning algorithms and SHAP analysis, the data was converted from categorical data (symbolic-based) to integer (numeric-based) data. To convert nominal data to numerical data in WEKA, the “NumericToNominal” filter was utilized for the data preprocessing. After converting these attributes, we can further execute classifiers, clustering techniques, or any other analyzing tools using the modified dataset in the WEKA environment and for the SHAP analysis using Python programming language.

#### 2.1.2. Dataset Balance

As the original dataset was unbalanced (15,306 negative cases and 1794 positive cases), the dataset was resampled by SMOTE. SMOTE is an oversampling algorithm the primary purpose of which is to amplify new minority specimens across a sample in which research can create more simulations to reach a balance among the different types of specimens. Generate new synthetic samples for the minority samples, select some nearby samples around the minority sample, randomly select a neighbor, and randomly generate one feature at a time within the distance between the two so that the two properties (positive and negative cases) are adjusted to the same amount.

#### 2.1.3. Model Validation

Cross-validation and percentage split are both techniques used in machine learning to assess the performance of a model on a dataset. This study used K-fold cross-validation to verify the trained model’s performance because it provides a more robust estimate of model performance through using multiple train-test splits. K-fold cross-validation also helps to mitigate the impact of dataset variability and reduces the risk of overfitting or underfitting to a particular subset of the data [23,24]. The entire dataset was first divided into K subgroups of equal size. A subset is used as validation data, and the remaining K-1 samples are used as training samples. Cross-validation is repeated K times, testing each specimen once. The output is the mean of all test results. As this method is time-consuming and computationally costly, we performed 10-fold cross-validation.

### 2.2. Artificial Intelligence with Various Machine Learning Algorithms

In the pursuit of risk factor analyses through the application of Artificial Intelligence techniques, our experimental approach involved the incorporation of renowned machine learning algorithms, namely, IBK, Random Tree, Decision Table, J48, and Random Forest, as recommended in the extant literature [10,11,12]. A brief introduction to these algorithms is shown below:

IBK is a k-nearest neighbor classifier, which determines the classification according to the number of closest distances to the case.

The Random Tree model is based on classes that consider randomly selected K attributes on each node.

The Decision Table model is a majority classifier for building and using simple decision tables.

The J48 algorithm uses various dichotomies to form a dendrogram to classify different glass types as a decision tree classifier.

The Random Forest algorithm combines multiple decision trees and randomly assigned training data to increase the final calculation result significantly. It can also be said to be a classification model composed of many different decision trees.

For the Random Forest algorithm with the best results, the folds are set to different correctly classified instances for comparison.

### 2.3. Interpretability

Artificial intelligence has sometimes been criticized by people for its black box problem since it is hard to explain the result it derived. Using the Random Forest and SHAP algorithms, this study primarily aims to observe and explain the significance of feature factors for the experimental results. The former is a single feature ranking method to observe the impact of model performance, while the latter plays the role of flexible observation of features related to model output. SHAP values are a method for explaining the output of machine learning models. They are based on cooperative game theory and calculate the contribution of each feature to the prediction. SHAP values provide a fair allocation of the prediction to each feature, considering all possible combinations.

SHAP uses a global interpretability graph, which determines the importance of all features according to positive or negative correlation coefficients and a color bar. The importance diagram provides a prosperous SHAP value and outputs the influence direction information of a single point in rich colors, which can help to gain critical insights quickly. Dependency plots help to show correlations and interactions between two features and SHAP value trends. Local interpretability diagrams, waterfall diagrams, are designed to offer the interpretation of a single instance.

### 2.4. Performace Evaluation Indicators

Numerous performance evaluation indicators or metrics have been proposed in the scholarly literature for artificial intelligence techniques or machine learning algorithms [25,26,27]. Most of them are implemented and are available in the WEKA environment that has been utilized in this study. Five performance evaluation indicators (TP rate, FP rate, ROC area, PRC area, and F1-score) were selected for model building to compare the results. TPs (true positives) indicate that the prediction and actual condition are positive. FPs (false positives) indicate that the prediction is positive, but the actual situation is negative. FNs (false negatives) indicate that the prediction is negative, but the actual situation is positive. TNs (true negatives) indicate that the prediction is negative and the actual condition is also negative. The performance evaluation indicators and some related performance evaluators are defined in more detail as follows:

Sensitivity (recall and true positive rate): This value indicates the ratio of predicted true positive cases out of all positive cases.

$$\mathrm{Sensitivity}=\mathrm{Recall}=\mathrm{TP}\;\mathrm{Rate}=\frac{TP}{\left(TP+FN\right)}$$

Specificity (1 − FP rate): This value indicates the ratio of predicted true negative cases out of all negative cases.

$$\mathrm{FP}\;\mathrm{Rate}=\frac{FP}{\left(FP+TN\right)}$$

$$\mathrm{Specificity}=(1-\mathrm{FP}\;\mathrm{Rate})=\frac{TN}{\left(FP+TN\right)}$$

Precision (PPV; positive predictive value): This value indicates the ratio of predicted true positive cases out of all predicted positive cases.

$$\mathrm{Presicion}=\mathrm{PPV}=\frac{TP}{\left(TP+FP\right)}$$

NPV (negative predictive value): This value indicates the ratio of predicted true negative cases out of all predicted negative cases.

$$\mathrm{NPV}=\frac{TN}{\left(TN+FN\right)}$$

Accuracy: this value indicates the correctness of the prediction of all cases:

$$\mathrm{Accuracy}=\frac{\left(TP+TN\right)}{\left(TP+FP+FN+TN\right)}$$

ROC (Receiver Operator Characteristics) area: ROC is the area under the Receiver Operator Characteristics curve that shows how the number of correctly classified positive cases varies with the number of incorrectly classified negative cases.

PRC (precision–recall curve) area: PRC is the area under the precision and recall curves and is a typical metric used to evaluate a model when facing unbalanced precision and recall data.

F-1 Score (Also called F-Measure): F-1 Score takes the average of the reconciliation of the accuracy rate and the recall rate. Its formula is shown below:

$$\text{F-1}\;\mathrm{Score}\;(\text{F-Measure})=\frac{2}{\left(\frac{1}{Precision})+(\frac{1}{Recall}\right)}$$

## 3. Results

### 3.1. Performance Evaluation

As described in the previous sections, artificial intelligence with five famous machine learning algorithms, namely, IBK, Random Tree, Decision Table, J48, and Random Forest, have been utilized in the WEKA environment for our experiments. The primary parameter settings for each machine learning algorithm have been summarized in Table 1.

Table 2 compares the results obtained with the different algorithms. The results from the Random Forest algorithm were the best (F1 value of 92.2%, ROC area of 96.8%, PRC value of 96.5%, and accuracy of 92.1%). In this case, the Random Forest algorithm significantly outperforms the other four algorithms, and it has been concluded that the main gains are due to the random design of the bootstrap subsampling and feature selection of split nodes. Random Trees are built by considering a class-building tree with K randomly picked qualities at each node, resulting in a more potent ensemble synergy.

WEKA was applied to these five artificial intelligence models for comparison, as illustrated in Table 2. The Random Forest algorithm had the largest ROC area (96.8%), indicating a significantly better TP rate and FP rate trade-off of the threshold setting. Since the Random Forest algorithm outperformed the others, more experimental results for the 5-fold, 10-fold, and 15-fold cross-validation are shown in Table 3.

### 3.2. Interpretability

Prior to delving into the interpretation of features, it is essential to realize that model interpretability is not always synonymous with causation. It is crucial to note that the SHAP values do not offer causation. Instead, they help us understand how the model reacts via data with various attributes. Due to the interpretability of the model, this study does not require black box fumbling, and the results can be interpreted in the analysis.

**Figure 2:**
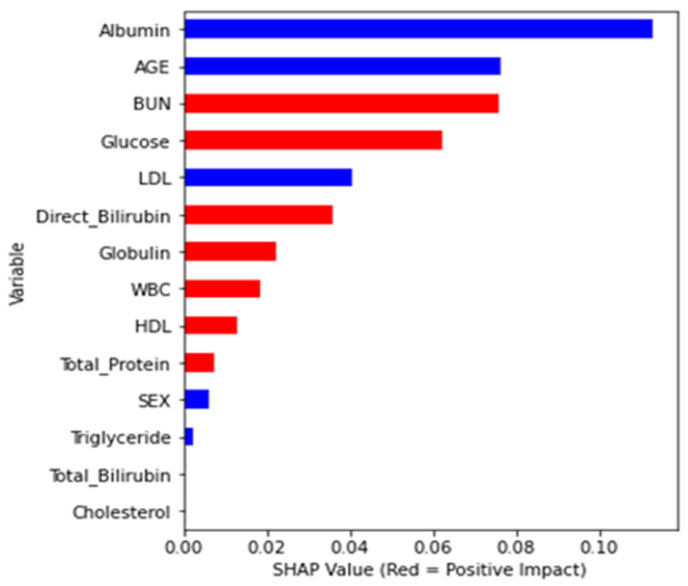
SHAP value and feature importance plot.

First, the Random Forest algorithm was used to determine the importance of the global features. Figure 2 shows that albumin, age, and BUN are the first three influencing characteristics. The SHAP value in the graph shows more information. Among them, albumin/age/BUN has the most crucial and positive effect, which means that a larger eigenvalue brings a greater probability of occurrence. However, albumin had the highest negative impact. In addition, increasing age is associated with the risk of CKD. In general, the red bar in Figure 2 has a positive correlation coefficient, while the blue bar has a negative correlation coefficient.

**Figure 3:**
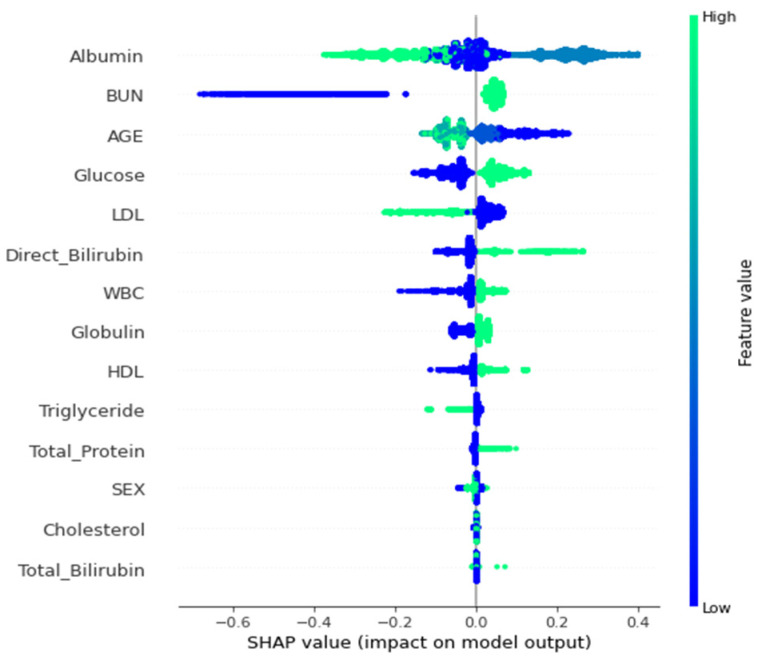
Bee swarm summary plot for feature importance.

**Figure 4:**
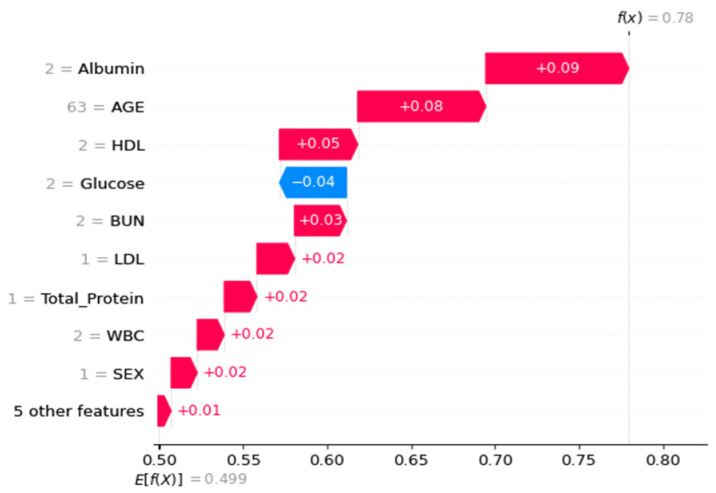
Waterfall diagram for local interpretation of specific parameters.

Next, SHAP was utilized to determine the contributions of all features (Figure 3). BUN and glucose were the features with higher eigenvalues (i.e., green dots contribute positive SHAP values). In contrast, the eigenvalues of attributes such as albumin, age, BUN, or triglycerides were negatively correlated (i.e., blue dots contributed negative SHAP values). Figure 4 illustrates the local interpretation of the positive impact factor of a single factor.

### 3.3. Extended Results for Dual-Attribute Factors

Based on the data percentiles, we converted the numerical variable “age” into three different nominal variables: “Age_1”, “Age_2”, and “Age_3”. Age_1 has two categories: age percentile less than 50%, and above 50%. Age _2 has three types: age percentile less than 25%, above 50%, and those in between. Age_3 has four categories: age percentile less than 25%, between 25 and 50%, between 50 and 75%, and above 75%.

Fifteen dual-attribute factors were derived by combining the top six single-attribute factors, as shown in Figure 2. The initial single-attribute factor and the resulting 15 dual-attribute factors were mixed for a second Random Forest analysis. The best outcomes were derived when the age was categorized in the manner of attribute “Age_3”.

**Figure 5:**
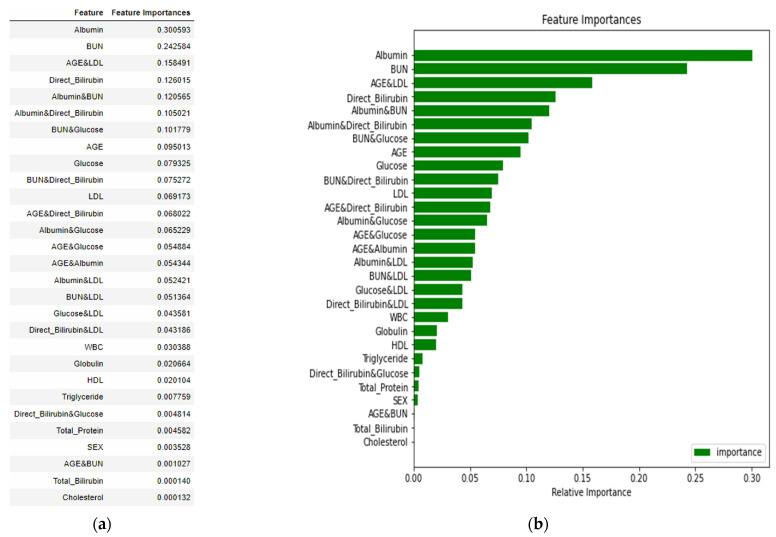
Feature importance plot of single- and dual-attribute factors. (**a**) importance value in descending order, (**b**) a bar chart of feature importance in descending order.

**Figure 6:**
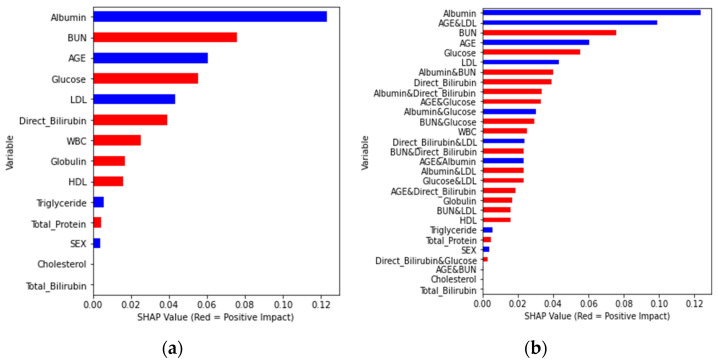
Feature importance plot of SHAP value for (**a**) single-attribute factor, (**b**) single- and dual-attribute factors.

Figure 2 represents the feature importance of a single-attribute factor presented by changing the numerical variable age to the nominal variable Age_3. Finally, the integration of single- and dual-attribute factors is shown in Figure 5. Moreover, the final analysis is shown in Figure 6a,b, in which red denotes an influencing element with a positive overall impact and blue denotes one with a negative overall impact.

**Figure 7:**
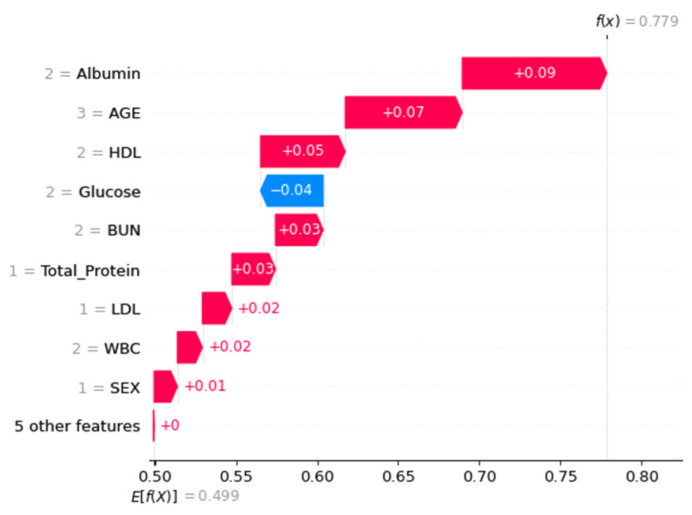
Waterfall plot using single-attribute factor examples for local interpretation.

**Figure 8:**
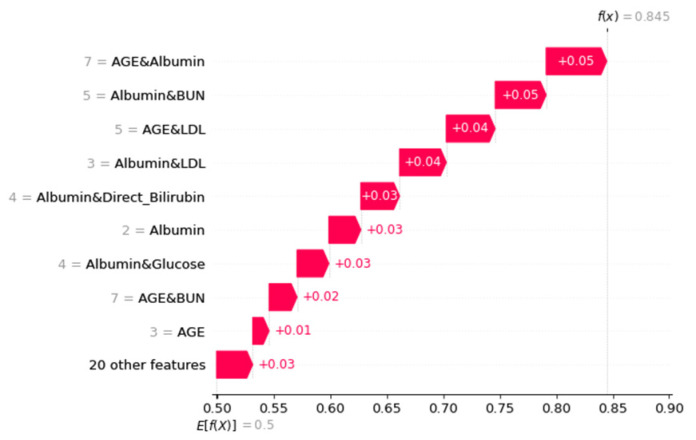
Waterfall plot using single- and dual-attribute factors instance for local interpretation.

For the Waterfall plot in Figure 7 and Figure 8, we used the same two random samples as in Figure 4. We anticipate using SHAP’s benefit of allowing various parties to see the same data in order to assess the layer-by-layer silk connection issue.

## 4. Discussion

The progression of chronic kidney disease (CKD) is multi-faceted and complex. As a result, the proper management of CKD to slow the progression of this disease is very important. Specifically, high-risk groups for CKD are patients with diabetes, high blood pressure, related family history, and old age [28,29]. CKD has an increased risk of heart and vascular disease. When CKD is not adequately managed, it may eventually lead to end-stage kidney disease, which may require dialysis or kidney transplantation. Determining the influencing factors or possible triggering factors could improve patient outcomes.

In this study, we used machine learning to explore the interaction of relevant factors on an unbalanced dataset. The Random Forest algorithm showed a better predictive ability than the other algorithms. With a 10-fold cross-validation on a balanced dataset, the model can achieve an overall accuracy rate of 92.1%, recall rate of 92.1%, ROC area of 96.8%, and F1 of about 92%. In addition, different folds are set to check whether the results of the different folds in the Random Forest algorithm are different. In folds 5, 10, and 15, even if there is no noticeable change in the ROC area, an accuracy of 92.13% is achieved in the 10-fold cross-validation.

The SHAP analysis in our study found that the top six influencing factors were serum albumin, BUN, age, direct bilirubin (D-Bil), glucose, and LDL, after removing creatinine. The serum albumin is the most influential factor in the progression of CKD among all other factors. BUN alone is the second most important risk factor in our research. Age is another risk factor for CKD. The prevalence of nephrosclerosis increases concomitantly with age, ranging from 2.7% among individuals below 29 years without comorbidities to 73% in healthy individuals aged beyond 70 years. [30]. In the meantime, the vessel formed between the afferent and efferent arterioles causes a shunt, especially at the juxtamedullary nephrons. The other arteries of the kidneys become thickened and lose self-nominal vascular reflection. The renal tubules display fatty degeneration and a thickened basement membrane. Age alone and LDL are also the most influential risk factors in our study. In addition, combining them as dual-attribute factors indicated that albumin and BUN and BUN and glucose have a higher impact than most single-attribute factors.

The results show that the SHAP analysis not only predicts but also classifies important characteristics of several classification combinations. It is convenient to use the same dataset to present in different ways, observe the possible interaction between factors from various aspects, explore the possible causes, and formulate corresponding countermeasures and in-depth discussions in the follow-up.

CKD is a multifactorial, complex condition that requires proper management to slow its progression, and therefore, it has attracted more attention from researchers for its risk prediction in recent years [31,32,33,34,35]. However, most studies focused on the factors predicted using various models from various methods or algorithms. In this study, not only were the essential elements of CKD found, but also the impact direction and strength of each factor were illustrated locally and globally using SHAP techniques. Nevertheless, the results of this study might be applied only in Thailand due to the nature of the data source. However, this limitation could be removed in the future, should more data be collected globally.

## 5. Conclusions

The most significant contribution of this study is its ability to predict risk factors for worsening conditions in CKD patients. In this study, artificial intelligence with five famous machine learning algorithms, namely, IBK, Random Tree, Decision Table, J48, and Random Forest, were deployed in the WEKA environment to develop a risk prediction model for CKD in Thailand. The experimental results of this study illustrated that the Random Forest algorithm outperformed the others, with a promising accuracy of 92.13% and ROC area of 96.8%. Furthermore, the risk factor prediction model, coupled with the interpretative insights afforded by the SHAP technique, exhibits considerable promise for its prospective utilization in developing personalized interventions and treatments. Consequently, the authors are strategically positioned to integrate these prediction algorithms and visualization techniques into a comprehensive system to advance future research endeavors.

## Figures and Tables

**Table 1:** Parameter settings for machine learning algorithms in WEKA.

Machine Learning Algorithms	Parameter Settings
IBK	KNN: 1, batchSize: 100, crossValidate: False, debug: False, distanceWeighting: No, doNotCheckCapabilities: False, meanSquared: False, nearestNeighbourSearchAlgorithm: LinearNNSearch, numDecimalPlaces: 2, windowsSize: 0
Random Tree	KValue: 0, allowUnclassifiedInstances: False, batchSize: 100, breakTiesRandomly: False, debug: False, doNotCheckCapabilities: False, maxDepth: 0, minNum: 1.0, minVarianceProp: 0.001, numDecimalPlaces: 2, numFolds: 0, seed: 1
Decision Table	batchSize: 100, crossVal: 1, debug: False, displayRules: False, evaluationMeasure: accuracy/RMSE, numDecimalPlaces: 2, search: BestFirst-D1-N5, useIBK: False
J48	batchSize: 100, binarySplits:F alse, collapseTree: True, confidenceFactor: 0.25, debug: False, doNotCheckCapabilities: False, doNotMakeSplitPoitActualValue: False, minNumObj: 2, numDecimalPlaces: 2, numFolds: 3, reuceErrorPruning: False, saveinstanceData: False, seed: 1, subtreeRaising: True, unpruned:False, useLplace: False, useMDLcorrection: True
Random Forest	bagSizePercent: 100, batSize: 100, reakTiesRandomly: False, calcOutOfBag: False, computeAttributeImportance: False, debug: False, maxDepth: 0, numDecimalPlaces: 2, numExecutionSlots: 1, numFeatures: 0, numIterations: 100, outputOutOfBagComplexityStatistics: False, printClassifiers: False, seed: 1, storeOutOfBagPredictions: False

**Table 2:** Classification performance of different algorithms.

Algorithm	TP Rate (Recall, Sensitivity)	FP rate (1-Specificity)	Precision	F1 Score	ROC Area	PRC Area	Accuracy (%)	Category
IBK	0.825	0.041	0.952	0.884	0.955	0.949	89.1546	Normal
	0.959	0.175	0.845	0.898	0.955	0.953		Abnormal
Random Tree	0.883	0.064	0.933	0.907	0.918	0.902	90.9839	Normal
	0.936	0.117	0.889	0.912	0.918	0.877		Abnormal
Decision Table	0.867	0.103	0.893	0.880	0.929	0.921	88.1909	Normal
	0.897	0.133	0.871	0.884	0.929	0.925		Abnormal
J48	0.885	0.074	0.923	0.904	0.926	0.906	90.5593	Normal
	0.926	0.115	0.890	0.907	0.926	0.896		Abnormal
Random Forest	0.907	0.065	0.934	0.920	0.968	0.960	92.1305	Normal
	0.935	0.093	0.910	0.922	0.968	0.965		Abnormal

**Table 3:** Random forest classification at various k values.

Folds		TP Rate (Recall, Sensitivity)	FP Rate (1-Specificity)	Precision	ROC Area	Prc Area	Accuracy (%)	Class
5		0.901	0.063	0.935	0.968	0.960	91.9154	Normal
		0.937	0.099	0.905	0.968	0.966		Abnormal
	Weighted Avg.	0.919	0.081	0.920	0.968	0.963		
10		0.907	0.065	0.934	0.968	0.960	92.1305	Normal
		0.935	0.093	0.910	0.968	0.965		Abnormal
	Weighted Avg.	0.921	0.079	0.922	0.968	0.963		
15		0.905	0.064	0.934	0.968	0.960	92.0394	Normal
		0.936	0.095	0.908	0.968	0.966		Abnormal
	Weighted Avg.	0.920	0.080	0.921	0.968	0.963		

## Data Availability

The data are available from the Research Ethic Committee No. 4 Faculty of Medicine, Chiang Mai University for researchers who meet the criteria for access to confidential data. Requests for the data may be sent to the Research Ethic Committee No. 4 Faculty of Medicine, Chiang Mai University.
